# Radon concentration in conventional and new energy efficient multi-storey apartment houses: results of survey in four Russian cities

**DOI:** 10.1038/s41598-020-75274-4

**Published:** 2020-10-22

**Authors:** Ilia V. Yarmoshenko, Aleksandra D. Onishchenko, Georgy P. Malinovsky, Aleksey V. Vasilyev, Evgeniy I. Nazarov, Michael V. Zhukovsky

**Affiliations:** Institute of Industrial Ecology, Ural Branch of Russian Academy of Sciences, S. Kovalevskoy St., 20, Ekaterinburg, Russia 620219

**Keywords:** Atmospheric chemistry, Energy and society, Risk factors

## Abstract

During last decades, energy saving in new buildings became relevant within the energy efficiency strategies in various countries. Such energy efficient building characteristics as air tightening and low ventilation can compromise indoor air quality, in particular, increase radon concentration. In Russia, a significant part of the new buildings is the energy efficient multi-storey apartment houses. The aim of this study is to assess the significance of possible radon concentration increase in new energy efficient buildings in comparison with typical conventional multi-storey houses of previous periods. Radon surveys were conducted in Russian cities Ekaterinburg, Chelyabinsk, Saint-Petersburg and Krasnodar. The radon measurements were carried out in 478 flats using CR-39 nuclear track detectors. Energy efficiency index (EEI) was assigned to each house. All buildings were divided into six main categories. The smallest average radon concentration was observed in panel and brick houses built according to standard projects of 1970–1990 (four-city average 21 Bq/m^3^). The highest average radon concentration and EEI were observed in new energy efficient buildings (49 Bq/m^3^). The trend of radon increase in buildings ranked with high EEI index is observed in all cities. The potential increase of radiation exposure in energy-efficient buildings should be analyzed taking into account the principles of radiological protection.

## Introduction

Indoor exposure to decay products of radon gas is recognized as a human carcinogen risk factor. Findings from epidemiologic studies support a strong causal association between indoor radon concentration and lung cancer mortality^1,2^. According to combine of several studies^3^ excess risk of lung cancer is about 14% per radon concentration 100 Bq/m^3^ linked to an exposure period of 25–30 years. ICRP consider that there is no known threshold concentration below which radon exposure presents no risk of lung cancer^4^. The health risk assessment puts radon in the second place after smoking among lung cancer causes^4^. Population protection against indoor radon is provided by control of natural radioactivity during construction and operation of buildings^5^. In this relation, geogenic radon potential and anthropogenic factors influencing indoor radon entry should be taking into account. An important aspect of the radon problem is the changes in construction technologies and impact of these changes on processes of radon accumulation in buildings.

Since the 1980s, in Russia and other countries of the world, energy saving and energy efficiency became important requirements for the transition to the principles of environmentally sustainable development. The global need to reduce energy consumption in different spheres of human activity is caused by several reasons. To achieve limiting global average temperature rise, a variety of measures are required to reduce greenhouse gases emissions^6^. High energy costs limit economic growth especially in developing countries. According to statistic data on household spending, heating costs make up a larger part of the expenditures in low income than in high-income families, reducing availability of education, medical care and other socially important needs^7^.

In the overall structure of energy consumption, a significant part is associated with housing and communal services—up to 40% in developed countries^8^. Reducing energy consumption and improving the energy efficiency of buildings is one of the objectives of the energy efficiency strategy in various countries (for Russian Federation see ref.^9^). The major potential of energy saving relates to decreasing of heat losses through building envelope (walls, windows, roof, etc.) due to thermal conductivity and convection. One of the efficient methods of limiting heat consumption is a significant reduction of uncontrolled air exchange with the outside atmosphere in passive mode of operation (without active regulation of the air exchange by dwellers). In Russia, the requirements on the energy efficiency of buildings have been established since 1996, including the regulation of heat saving, heat consumption and other parameters^10^. The energy efficiency class of a building in Russia is determined by the value of the energy consumption for heating, ventilation and hot water supply^11^. The European Union has adopted the Energy Performance in Buildings Directive (DIRECTIVE 2010/31/EU) which obliges to construct only near zero energy buildings (defined as high energy efficiency buildings in a broad sense) in European countries by 2021.

Changing the energy characteristics of a dwelling that reduce heat loss can affect indoor air quality. In particular, the building air tightening may inhibit radon and other pollutants from leaving the indoor environment and cause it to accumulate^12,13^.

Increase in indoor radon concentration after reconstruction in order to reduce heating loss was studied in different countries. Several times increase of radon concentration was revealed in certain single-family houses after installation of new PVC windows in regions of high geogenic radon potential in the Czech Republic^14^. In France, technical measures to increase heat saving were undertaken in more than a half of buildings, which resulted in the increase of geometric mean of radon concentration by 1.6^15^. Also, the growth of radon concentration was observed in retrofitted houses in Switzerland^16,17^ and Germany^18^. In England, measures to reduce the permeability of the shell of single-family houses led to a decrease in uncontrolled ventilation by an average of 1.9 times^13^ and an increase in radon concentration by about 1.7 times^19^. Comparative studies in samples of energy retrofitted and unreconstructed residential low-rise buildings demonstrated 22–120% increase of average of radon concentration^20^. Studies in the Czech Republic^21^ and Russia^22^ have shown 1.6 times average increase of radon concentrations in children institutions. In the United States, the increase of radon concentration by 33% (to 118 Bq/m^3^ on average) was observed in houses included in the national energy saving program in the zone with elevated geogenic radon potential; at the same time, in the zone with the lowest radon potential, the radon concentration did not change (30 Bq/m^3^)^23^. Similarly, in retrofitted houses in rural settlements on the Techa River, Russia located in the geological zone of low soil permeability, the radon concentration increased by 50% to 135 Bq/m^3^ on average; in the geological zone with high permeability, the average radon concentration was the same^24^. In Lithuania and Finland, radon concentrations in multifamily buildings that were retrofitted with an increase in energy efficiency class and unreconstructed buildings were compared^25^. According to the results of the analysis, an increase in the radon concentration was observed in Lithuania after increase of energy saving by 1.6 times to 44 Bq/m^3^^25^. In Finland, radon concentration did not change in apartment buildings with mechanical ventilation (74 Bq/m^3^)^25^. Recent study in Ireland and the UK showed decreasing of radon concentration in two storey houses with mechanical ventilation (passive houses)^26^. In general, the change of radon concentration depends on the conditions of entry, ventilation pattern and relative contributions of the diffusion and convective mechanisms of radon transport^20^.

Energy saving in new buildings is one of the main points of energy efficiency strategies in various countries. In new houses built to meet energy efficiency requirements, increase in radon concentration compared to low-energy efficiency classes houses is expected as well. The influence of energy-savings measures on indoor radon concentration in new buildings were found in several studies in different countries. In the United States (New York), radon survey conducted in conventional and energy-efficient single-family houses in the early 1980s have shown that radon concentration was almost three times higher in a sample of houses with airtight envelope than in other houses^27^. In Canada significant increase in radon due to energy efficiency measures was observed when buildings were grouped into year-of-construction categories^28^. In Romania, measurements in one-story buildings, constructed to meet the energy saving requirements since 2000 shown average radon concentration 27% higher than in conventional houses^29^.

In Finland, measures to increase the energy efficiency of buildings have been applied since the 1980s. Later a requirement for equipping buildings with mechanical balanced and extraction ventilation was included to the building codes for radon prevention purposes. Since 2006, most of new single-family houses have met both the energy efficiency and radon protection requirements. Such approaches to regulation in construction resulted in corresponding changes in the accumulation of indoor radon: average radon concentration was 104 Bq/m^3^ in houses built in 1955–1964, by the end of the 1990s this value increased to 150 Bq/m^3^, in buildings constructed in 2006–2008, the average radon concentration decreased to 95 Bq/m^3^^30^. Similarly, relatively lower radon concentration in energy efficient houses equipped with mechanical balanced and extraction ventilation was observed in Norway^31^, Austria^32^ and Switzerland^33,34^.

A significant part of the housing stock in Russian cities is composed of multi-storey apartment houses equipped with natural ventilation systems. A common technical scheme of ventilation implies that fresh air enters the rooms through leaks in the building shell and stale air is removed by vertical ventilation channels through ducts usually arranged in the kitchen rooms, bathrooms and toilets of flats. Air movement occurs due to the temperature difference between the external and internal air and wind pressure. In Russia, the requirements for the construction of energy-saving buildings have been introduced since the late 1990s. In 2017, more than 75% of approximately 9000 new apartment buildings put into operation were assigned to high energy efficiency classes B, A, A+ , A++^9,11^. Reducing energy consumption in new multi-storey apartment buildings is achieved by such architectural and construction solutions as use of monolithic concrete and reinforced concrete structures in combination with wall insulations and sealed double-glazed windows, as well as special building design reducing the uncontrolled air exchange. Due to low air leaks through the external building envelope, natural ventilation systems supply insufficient amount of the fresh air and doesn’t guarantee normative air exchange in new Russian energy efficient multi-storey apartment buildings (0.35 h^-1^)^35^.

Until present study, surveys of radon accumulation in new energy-efficient buildings in Russia were carried out only in Ekaterinburg^35–38^. As a part of these studies, a low air exchange rate in new energy efficient mid and high-rise buildings (> 9 floors), about 0.1–0.2 h^−1^, and the effect of low air exchange rate on radon concentration in flats were observed. The example of Ekaterinburg demonstrated the relevance of studying the problem of radon accumulation in flats of new energy-efficient large buildings.

The aim of this study is to assess the significance of possible increased accumulation of radon in modern multi-storey apartment energy efficient houses in various regions. The study was conducted on a sample of four large Russian cities situated in different climatic and geographical zones. The study includes radon surveys in samples of flats in old and new buildings of various energy efficiency classes.

## Materials and methods

### Region of study

**Figure 1:**
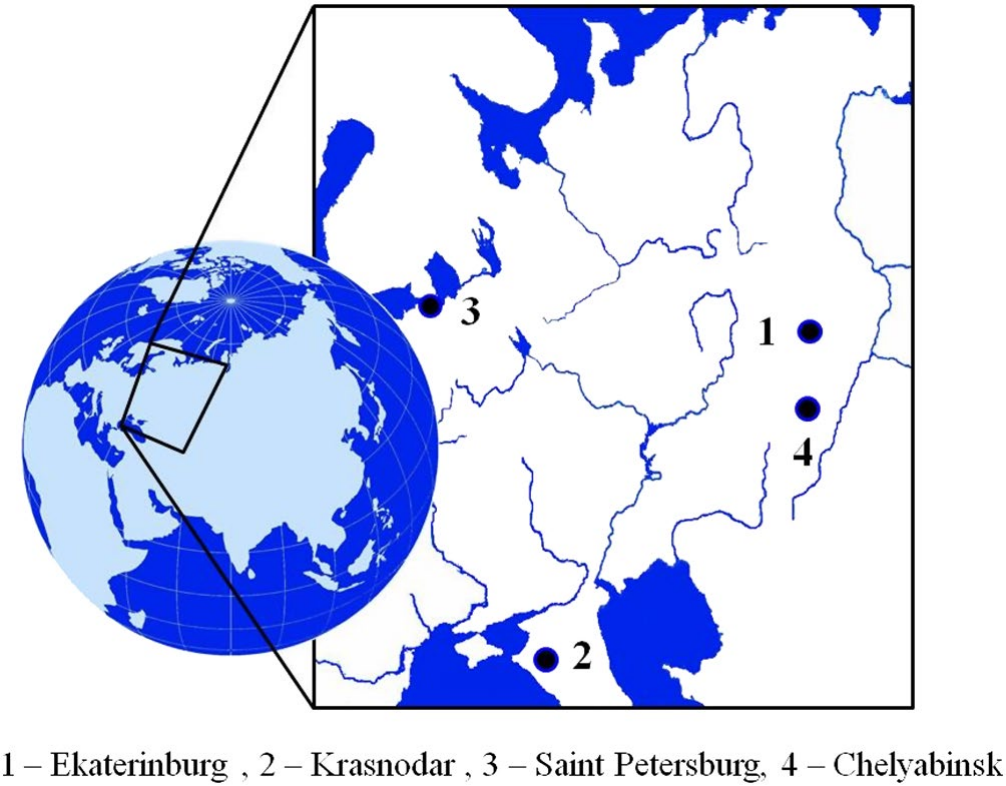
Location of the surveyed cities (map services and data available from U.S. Geological Survey, National Geospatial Program, https://viewer.nationalmap.gov/advanced-viewer/).

Four Russian cities, located in different climatic and geographical zones were selected to conduct radon survey: Ekaterinburg, Chelyabinsk, Saint-Petersburg and Krasnodar (Fig. 1). Each city is a large administrative centre with a population of about or more than a million people where high rates of building construction were achieved in recent decades: 20–35% of contemporary residential housing stock was built since 2000. Population and general climatic characteristics of the surveyed cities are presented in Table 1.

**Table 1:** Population and climatic conditions of the surveyed cities.

City	Population in 2019, × 10^6^	Climate	Average annual temperature, °C	Average temperature in January, °C	Average temperature in July, °C
Ekaterinburg	1.48	Temperate continental	3.0	− 12.6	19.0
Krasnodar	0.92	Temperate continental /subtropical	12.1	0.6	24.1
Saint Petersburg	5.38	Temperate continental /moderately marine	5.8	− 5.5	18.8
Chelyabinsk	1.20	Temperate continental	3.2	-14.1	19.3

### Radon surveys

In each city indoor radon concentration measurements were performed in two samples of houses. The first sample included flats in new multi-storey apartment houses which supposed to be assigned to high energy efficiency class according to the Russian classification. The second sample was formed from multi-storey apartment houses built before 2000 for which low energy efficiency is expected.

In Ekaterinburg, samples were formed from the data base of the results of previous studies carried out as part of the other research projects realized in Institute of Industrial Ecology in 2015–2017. The entries with the same indoor radon measurement method and exposure duration were included to the current study. However the results were obtained in different seasons (winter—75%, spring/fall—25%).

In cities of Chelyabinsk, Saint Petersburg and Krasnodar the samples were formed within the frame of this research basing on approaches as follow:

1. invitation of volunteers from online communities of residents of buildings belonging to one of the required types;
2. offering participation in the study to members of organized groups, such as staffs of research and educational institutions;
3. using personal contacts.

To disseminate information about the study and attract volunteers to participate, a special group in popular Russian social network was established under supervision of research team members. Also a website was created to provide more detailed information about the study.

The research involved human participants. All experimental protocols were approved by Research Council of Institute of Industrial Ecology of Ural Branch of Russian Academy of Sciences. All methods were carried out in accordance with relevant guidelines and regulations. Informed consent was obtained from all participants (no subjects under 18 were invited).

The measurements of radon concentrations were carried out using CR-39 type RSKS nuclear track-etch detectors (RadoSys, Hungary)^39^. Radon detectors were calibrated both by the producer and within the framework of internal laboratory control of the Institute of Industrial Ecology. RadoSys regularly participate in intercomparison of radon measurements with RSKS detectors, in particular, recently, in international comparison of radon/thoron measurement instruments^40^ and in the framework of MetroRADON Project^41^. In the Institute of Industrial Ecology, the calibration coefficients provided by producer were verified by exposure of radon detectors in a radon chamber equipped with a radon source and radon-monitor AlphaGUARD PQ2000 PRO as a reference device, which was previously calibrated with using a NIST radon emanation standard.

Two detectors were installed in each flat. In flats with more than one room, the radon detectors were placed in living room and bedroom. The radon detectors had to be placed on any open surface at a height of 1–2 m and at a distance of more than 1 m from doors and windows. Detailed guidelines on handling with radon detectors were developed for participants of the study. Some of the volunteers attended a special popular science lecture with information about the study, organized by members of scientific team in home cities of participants. The participants of study were asked to fill out a questionnaire about their flats by themselves. The general information obtained by means of self-filled questionnaire included address, floor of the flat, type of room, the number of dwellers etc. Considering the objectives of the study, data on the type of the ventilation, frequency of active room ventilation, and type of windows were asked in addition to the characteristics usually recorded in radon surveys. Some essential information about the houses provided by participants of the study was verified applying official technical records.

Chemical etching after exposure and tracks reading was performed in the Institute of Industrial Ecology UB RAS using the automatic RadoSys equipment. An average flat radon concentration was estimated using results of two measurements.

Radon detectors were exposed for an average of three months. Three months measurements period instead of all year measurements was selected due to reasons as follow. Considerable part of volunteers perceived three months experiment acceptable for the participation. Longer period could result in low rate of agreement to participate and decrease percentage of radon detectors returned to the laboratory. According to previous study such duration of radon detector exposure provides satisfactory uncertainty of indoor radon concentration results^42^. Considering known average month temperature it is possible to select measurements period with mean temperature close to an annual average value. Estimation of the annual average radon concentration was not the task of the study, while radon concentration obtained during few months period with the same outdoor temperature allow comparing results in different houses of the same region. The measurements in Chelyabinsk, Saint Petersburg and Krasnodar were carried out from August to November 2019, from September to December 2019 and from October 2019 to January 2020 respectively. Most of detectors were distributed and collected by the members of research team of this study. In some cases, radon detectors were distributed through the participants of the study, who involved their relatives or friends. In each city radon detectors were exposed simultaneously in flats of both samples.

Presence of thoron gas and other potential indoor air pollutants were not considered in this study.

### Energy efficiency index

To analyze association between achieved degrees of energy efficiency and measured indoor radon concentration a method of quantitative estimation of energy efficiency potential of a building was developed basing on the findings of the previous radon studies in Ekaterinburg^22,36–38^, where the role of architectural and construction solutions in radon entry and accumulation indoor was demonstrated.

To systematize the variety of architectural and construction factors determining the energy efficiency of buildings and characteristics of ventilation in multi-storey apartment house an expert evaluation approach was applied. All the factors were divided into two main categories:

1. architectural and planning (characteristics of building envelope, airtightness, area of external walls, glazing area, heat saving characteristics of vestibules, heat thermal protection characteristics of window systems etc.);
2. technical and technological (ensuring air exchange with minimal heat loss due to a mechanical supply and exhaust system with heat recovery, rational use of heat and energy sources at home).

Technical information about Russian multifamily residential buildings is available at open online databases of the foundation established for supporting communal services reform in the Russian Federation. The databases contain general construction characteristics of housing units (year of construction; type of building construction; number of floors; number of flats; residential/non-residential area, etc.), information about the structural elements of the houses (type of foundation; floor structure; material of walls, etc.) and engineering systems (electrical power system, ventilation system, garbage chutes, etc.). To analyze the architectural layout, we used photographed materials found at street view services of Google and Yandex and floor plans if available.

To quantitatively estimate energy efficiency potential of a building, energy efficiency index (EEI) was developed. The EEI provides combined estimate of the construction, architectural and technical characteristics of a building related to energy saving. The value of the EEI is the sum of the points estimated by some criteria presented in Table 2. Based on the information collected for studied buildings, an individual EEI were assigned to each housing unit.

**Table 2:** Evaluation of EEI.

Criterion	Description	Points estimation procedure
Architectural and planning max 1.4 points		
V1—Residential/non-residential area ratio	Equals to the ratio of the residential area, S_R_, to non-residential area, S_NR_, in a house	if S_R_/S_NR_ > 0.5: V1 = 0.5 if S_R_/S_NR_ ≤ 0.5: V1 = S_R_/S_NR_
V2—Airtightness	Depends on the type of facade walls:	
	highly efficient thermo insulated materials	V2 = 0.5
	brickwork	V2 = 0.25
	thermo insulated concrete panel	V2 = 0.125
	uninsulated facade of the building	V2 = 0
V3—Energy-efficient planning	Presence of heat saving tambours, stairwells and elevators:	
	yes	V3 = 0.25
	no	V3 = 0
Technical and technological max 0.5 points		
V4—Supply lines	Inversely proportional to the number of communication inputs to the house per 2000 m^2^ of the total area of the building	$V4 = \left( {\frac{{4 \cdot n_{4} }}{S} \cdot 2000 + 1} \right)^{ - 1} ,$ where n_4_ is the number of inputs, S is the total area of the building, m^2^
V5—Garbage chutes	Inversely proportional to the number of garbage chutes in the house per 2000 m^2^ of the total area of the building	$V5 = \left( {\frac{{4 \cdot n_{5} }}{S} \cdot 2000 + 1} \right)^{ - 1} ,$ where n_5_ is the number of garbage chutes

## Results

For radon surveys in Chelyabinsk, Saint Petersburg and Krasnodar, sets of two radon detectors were given out to the 430 participants. The percentage of returned detectors in different cities was in the range 78–92%. A part of the distributed detectors either were not returned by the participants for various reasons or were exposed in detached and other types of single family houses. The radon detectors were returned to the laboratory from 375 participants living in the multi-storey apartment houses. The Ekaterinburg samples size chosen to include to this study was 103 flats. Thus totally results of radon measurements in 478 dwellings in the multi-storey apartment houses were available for the further analysis.

The average temperatures for the radon detectors exposure period in different cities were as follow:

**Table:** 

Ekaterinburg	– 4.7 °C,
Krasnodar	5.0 °C
Saint Petersburg	5.6 °C,
Chelyabinsk	8.8 °C

As can be seen from these data and Table 1, the difference between the average temperatures for the measurement exposure period and the corresponding average annual temperatures is from 0.2 to 7.7 °C. This difference is less than 20% of the temperature difference of the coldest and warmest months of the year. Taking into account the relatively small difference between temperature during measurements and average annual temperature, it was decided to avoid additional seasonal normalization.

Based on the questionnaires and analysis of architectural and construction factors information about the surveyed buildings, the entire housing stock was divided into the following main categories.

1. Brick buildings with 2–5 storeys. Houses of this type were built mainly in 1930–1960. The main building material is brick; floor panel is made of reinforced concrete or wood. The façade walls are partially or fully plastered. The ventilation system is natural. Exhaust ventilation ducts are installed in the kitchen and bathroom. Continuous vertical stairwells are designed from the house entrance to the last floor. This group includes houses, which, together with similar panel five-story buildings, were the main type of buildings of the period of mass housing construction in 1950–1960.
2. Panel 5-story buildings. Houses of this type were built in 1950–1970. The walls and floor panels are made of prefabricated reinforced concrete panels. The ventilation system is natural, the kitchen and bathroom are equipped with an exhaust ventilation ducts. Vertical continuous stairwells are designed.
3. Panel buildings 7–12 storeys. This type of houses was used in the block development in 1970–1980. The walls and floor panels are made of prefabricated reinforced concrete panels. The stairwell represents a continuous volume over the height of the building including elevator halls. Often there is a garbage chute. Natural ventilation is a supply or exhaust type or combined type.
4. Brick buildings with more than five storeys. This type of houses was built mainly in 1970–1980. Floor panels are made of reinforced concrete. The stairwells also represent the continuous volume over the height of the building; the elevator halls are connected to stair halls. There is the garbage chute in some houses. The walls are plastered or tiled. The ground floor is often reserved for non-residential premises.
5. Panel buildings with more than ten storeys built in the period from 1990s to the present time. This type of houses is differing from the panel multi-storey buildings of earlier years of construction by planning decisions. In particular, stair halls are separated from the stairwells by tambours. The elevator halls may be separated from stair halls as well.
6. Modern energy efficient buildings over eight floors. This type includes buildings built in the period from 2005 to the present applying energy-saving architectural solutions and construction technologies. The PVC windows are used in all premises of the building, loggias are glazed. The façade walls are additionally insulated. Ground floor vestibules, external stairwells (fire escapes), and internal elevators halls are equipped with tambours and doors closers. Heat loss reduction is mainly achieved through structural and space-planning solutions: energy efficient house layout, continuous heat-insulated building shell, lack of thermal bridges, optimal glazing area, using window systems with a high level of thermal protection, the presence of vestibules with tambours at the entrances, etc.

In addition to the listed categories, some other types of buildings were included to the study. In Saint Petersburg, old multi-storey brick houses constructed before period of the World War I, October Revolutions and the Russian Civil War (1914–1922) contribute significantly to the contemporary housing stock. In Saint Petersburg, Ekaterinburg and Krasnodar the measurements were carried out in several low-rise (five storeys) modern energy-efficient buildings.

Important distinctive feature of the modern energy efficient buildings is the high number of floors (average is 18). Among the energy efficient buildings, there are significant amount of the high-rise buildings (81% is taller than 15 storeys). As can be seen in Table 3 average floor of flat, where radon concentrations measurements were performed, is much higher in this type of building than in others. According to analysis of self-reported information from dwellers, such characteristics as average number of dwellers in a flat, smoking prevalence among dwellers, and room ventilation preferences are approximately the same in different types of buildings (Table 3).

**Table 3:** Characteristics of flats by the main categories of the buildings.

Type of buildings	Average flat’s floor*	Average number of dwellers	Part of flats of no-smokers	Part of flats with reported permanent intentional ventilation
Brick 2–5 floor	3	2.3	0.73	0.24
Panel 5 floor	3	2.6	0.82	0.28
Panel 7–12 floor	5	2.7	0.83	0.37
Brick over 5 floors	6	2.2	0.63	0.33
Panel 1990-present	7	2.6	0.69	0.33
Energy efficient	11	2.7	0.76	0.28

*Ground level is counted as the first floor.

Based on the results of the analysis of the architectural and construction characteristics of buildings, the EEI of various types of high-rise buildings were determined.

To present the results of the radon survey the parameters of the lognormal distribution of the indoor radon concentration (flat average) were calculated in each group of buildings: the geometric mean (GM), the standard deviation of the logarithm (σ_LN_), as well as the arithmetic mean (AM) and the estimate of 95th percentile of the lognormal distribution. For each group of buildings the average EEI was calculated. The results of radon surveys and EEI estimation in four cities by types of buildings are presented in Table 4.

**Table 4:** Results of indoor radon concentration measurements and EEI estimations for different types of buildings in four Russian cities.

Type of buildings	N*	AM, Bq/m^3^	GM, Bq/m^3^	σ_LN_	95th percentile Bq/m^3^	EEI
Ekaterinburg						
Brick 2–5 floor	11	39	28	0.97	138	0.76
Panel 5 floor	15	36	29	0.63	82	0.40
Panel 7–12 floor	13	25	21	0.59	55	0.38
Brick over 5 floors	12	28	26	0.41	52	0.71
Panel 1990-present	16	34	29	0.60	78	0.44
Energy efficient	35	82	66	0.67	199	1.22
Low-rise energy efficient	1	16	16	–	–	0.86
Chelyabinsk						
Brick 2–5 floor	17	36	32	0.49	70	0.77
Panel 5 floor	16	27	25	0.38	47	0.42
Panel 7–12 floor	17	21	20	0.27	32	0.35
Brick over 5 floors	3	19	19	–	–	0.76
Panel 1990-present	62	25	23	0.39	45	0.57
Energy efficient	14	32	29	0.47	62	1.05
Saint Petersburg						
Before 1917	14	12	10	0.65	30	0.76
Brick 2–5 floor	14	26	21	0.67	63	0.68
Panel 5 floor	9	23	21	0.47	45	0.45
Panel 7–12 floor	6	18	16	0.43	33	0.46
Brick over 5 floors	16	15	14	0.35	25	0.69
Panel 1990-present	23	17	15	0.45	32	0.47
Energy efficient	62	31	27	0.57	68	1.21
Low-rise energy efficient	8	38	37	0.24	55	0.90
Krasnodar						
Brick 2–5 floor	10	42	36	0.53	86	0.74
Panel 5 floor	10	41	34	0.62	94	0.58
Panel 7–12 floor	7	21	20	0.36	36	0.36
Brick over 5 floors	6	19	18	0.34	32	0.78
Panel 1990-present	24	20	18	0.45	38	0.55
Energy efficient	36	28	27	0.29	43	1.25
Low-rise energy efficient	1	29	29	–	–	0.75

*N is number of measured flats.

**Figure 2:**
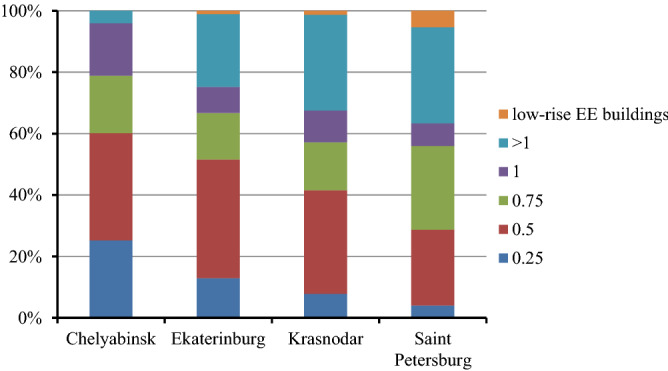
The share of buildings with different EEI by cities.

Figure 2 shows the distribution of buildings with different EEI in four cities. To draw this figure buildings were combined into 5 groups: 0–0.375; 0.376–0.625; 0.626–0.875; 0.876–1.125 and above 1,125, with EEI central values 0.25, 0.5, 0.75, 1, and > 1 respectively. For low-rise energy efficient buildings EEI was not calculated, as the methodology for EEI assessment has been developed for multi-storey buildings (mid-rise and high-rise). As can be seen from Fig. 2 the largest percentage of energy efficient buildings was observed in Saint Petersburg, the smallest—in Chelyabinsk. Also the largest share of low energy efficient buildings is observed in Chelyabinsk.

**Figure 3:**
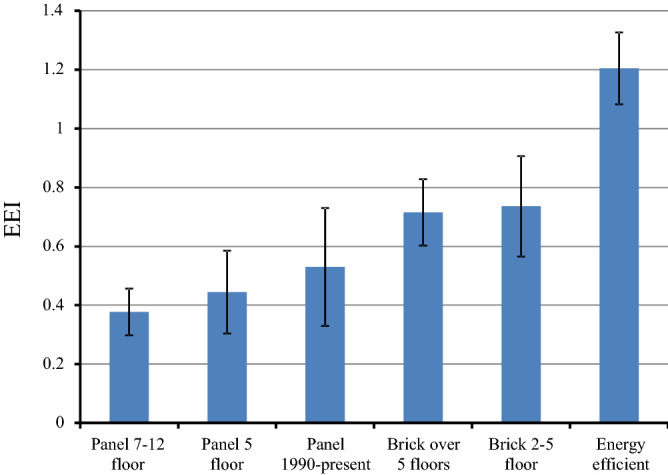
The average EEI by type of buildings, whiskers—standard deviation.

Figure 3 shows the difference in EEI between groups of typical buildings. The highest EEI is reasonably observed for the energy efficient buildings. Of the buildings of other types, the brick buildings are more energy efficient, while the EEI in this group is almost two times lower than in new buildings with high energy efficiency. The lowest energy efficiency is observed in panel buildings, almost three times less than in new energy efficient buildings.

**Figure 4:**
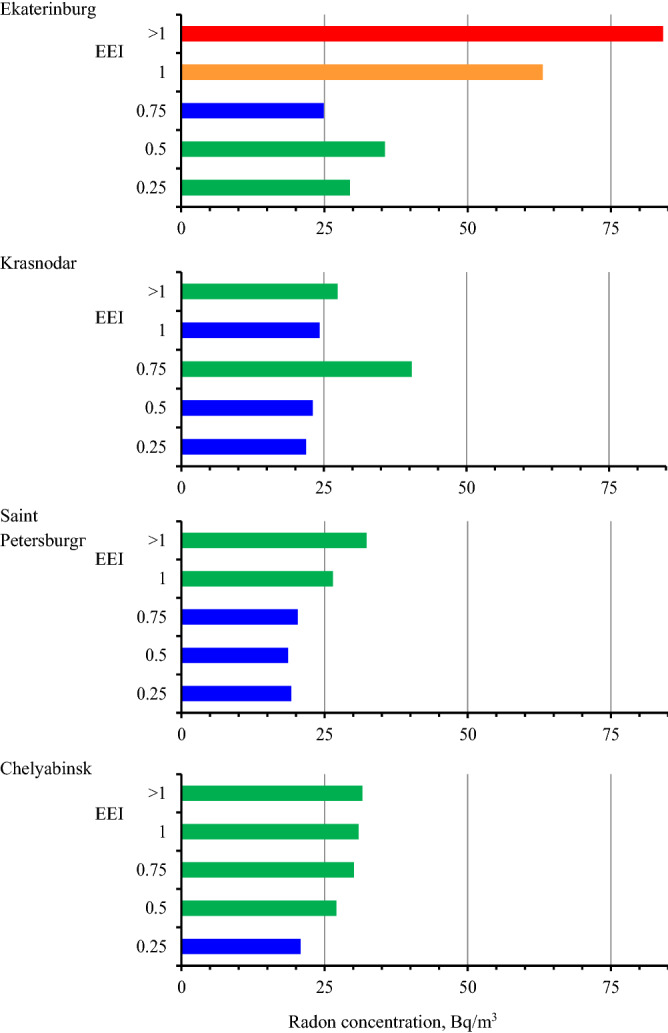
Average radon concentration in groups of buildings depending on EEI.

The AM of radon concentration were calculated in groups depending on the EEI in each of four cities (Fig. 4). In all cities, except Krasnodar, the maximum of radon concentration is observed in new energy efficient buildings. A general trend of increase in the average radon concentration with an increase in energy efficiency can be noticed in all cities.

**Figure 5:**
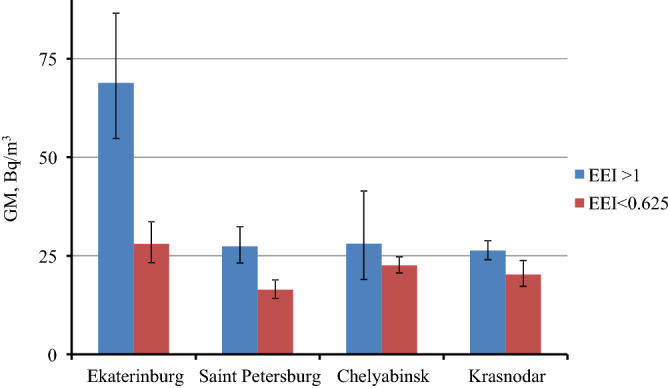
Geometric mean radon concentration in buildings of high and low energy efficiency classes with 95% CI.

Figure 5 presents a comparison of GM of radon concentration in buildings with maximum and minimum energy efficiency (groups of buildings with EEI > 1 and combined groups 0.25 and 0.5) with estimation of confidence intervals. In three out of four cities (Ekaterinburg, Saint Petersburg and Krasnodar), there is a statistically significant excess of GM of radon concentration in new energy efficient buildings.

**Figure 6:**
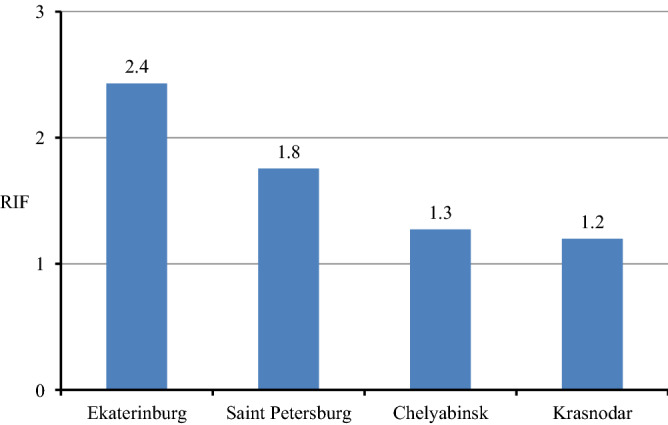
Radon increase factor in different cities.

In order to analyze change of radon concentration with implementing energy efficiency in new constructions, a radon increase factor (RIF) was calculated as ratio of AM of radon concentration in buildings with EEI > 1 to EEI < 0.625. As seen in Fig. 6, the RIF is in the range from 1.2 to 2.4 in different cities and the maximum of radon concentration growth was observed in Ekaterinburg.

**Figure 7:**
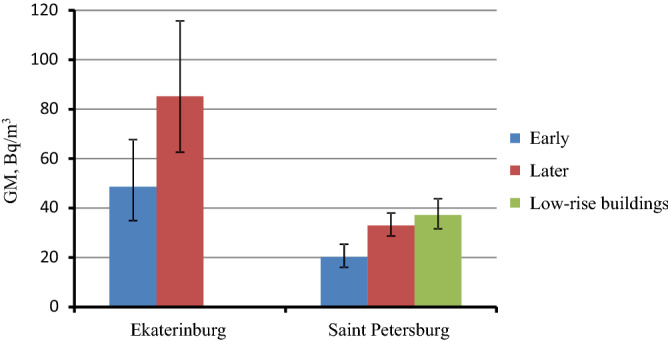
Geometric mean radon concentration in subgroups of energy efficient buildings depending on the year of construction: Ekaterinburg—earlier (number of measured flats N = 15) and later 2010 (N = 12); Saint Petersburg—earlier (N = 27) and later 2015 (N = 35). Low-rise energy buildings in Saint Petersburg—all the years of construction (N = 8). Whiskers—95% CI of geometric mean.

**Figure 8:**
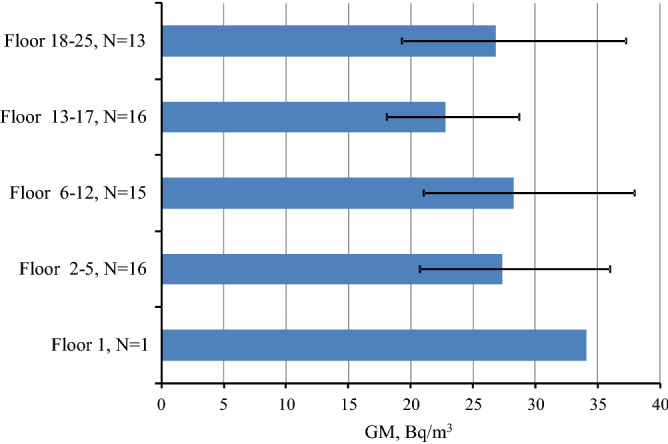
Geometric mean radon concentration depending on the floor in energy efficient buildings in Saint Petersburg (with 95% CI, N is number of measured flats).

In Ekaterinburg and Saint Petersburg the largest samples of new energy efficient buildings were obtained, which allowed more detailed analysis of factors affecting the accumulation of radon in this type of houses. As shown in Fig. 7 significant influencing factor is year of the building construction. In new energy efficient buildings built later (in Ekaterinburg after 2010, in Saint Petersburg after 2015) radon concentration is significantly higher than in buildings constructed earlier. On the example of Saint Petersburg a relatively higher radon concentration in low-rise energy efficient buildings can be observed. The dependence of radon concentration on the floor number in energy efficient buildings in Saint Petersburg are presented in Fig. 8. In this type of building only one measurement was performed on the first floor (floors are numbered applying Russian system with the ground floor being floor 1). For other floors there is no significant difference in GM radon concentration.

## Discussion

In the frames of this study, the indoor radon concentration was measured in various types of Russian multi-storey apartment houses with different energy efficiency characteristics using the same method. Radon detectors were exposed simultaneously in all types of buildings in each city, except Ekaterinburg.

There are two basic approaches to seasonal correction of indoor radon concentration measurements. The seasonal adjustment use known seasonal correction coefficients estimated during special studies performed in the region (such studies were not performed for each climatic region of Russia). The temperature normalization, another approach, suggests to estimate an annual radon concentration depending on the difference between temperature during measurement period and annual average value. The temperature of the outdoor atmosphere during exposure of detectors in flats approximately corresponded to the annual average temperature, therefore, the measured concentrations were considered as close assessment of the annual values. The chosen approach to seasonal correction should be considered as the source of uncertainty for comparison of indoor radon concentration between the cities. To analyze the radon levels in different types of buildings, it is essential to perform measurements simultaneously.

Application of two RSKS radon detectors provided relatively low range of σ_LN_ estimations (< 1, Table 4) for any subsample grouped by type of building. Low scatter of radon concentration within homogeneous groups (with regard to the type of building) increased a significance of comparison of radon concentrations between the groups.

A comparative analysis of radon concentrations and EEI in typical flat houses of mass development in Russia in the twentieth century and new multi-storey apartment buildings carried out in four Russian cities have shown the following results. The smallest average radon concentration is observed in panel and brick houses with a number of floors above five and built according to standard projects of 1970–1990 (Table 4). In these types of buildings, the average radon concentration in all cities was about 21 Bq/m^3^. Relatively higher levels of radon concentration are observed in low-rise (2–5 floors) buildings of the 1930–1960 construction period. The average radon concentration was 33 Bq/m^3^ in these buildings and it differs insignificantly across the surveyed cities. The relative decrease in energy efficiency in panel buildings of the 70–90 s compared with brick houses built before 1990 is caused by the material of the internal walls, the technologies used and the quality of work (poorly insulated joints of reinforced concrete slabs), the presence of garbage chutes, a large number of supply and service lines, the lack of tambours separating stairs and elevators.

The highest average radon concentration is observed in new energy efficient buildings (four-city average 49 Bq/m^3^). Higher energy efficiency in houses built after 2000 was achieved mostly due to increased airtightness of building envelope applying such technical measures to reduce or prevent thermal bridging as continuous insulation materials, double glazed PVC windows, large area of glazed loggias, etc. Decreased air permeability of building envelope significantly reduces the contribution of fresh air to the general air exchange of residential rooms, which leads to increased radon accumulation.

Both in low and high energy efficiency houses, thermal comfort is controlled by dwellers, in particular, by regulation of the air exchange (opening and closing windows and ventilation ducts). The EEI doesn’t take into account for living habits of dwellers however it reflect lower uncontrolled ventilation rate in energy efficient buildings. It can be supposed that controlled ventilation is lower by average value and duration in low energy efficient houses due to satisfactory ventilation rate without windows opening.

The general trend of average radon concentration increase in groups of buildings ranked with high EEI index is observed in all cities. Such trend may be characterized by the RIF (Fig. 6). While the buildings in the northern and southern regions of Russia principally have the same construction and architectural solutions undertaken to reduce the heat loss, and the average outdoor temperature during the measurements was also approximately the same, close values of RIF may be expected. However RIF varies noticeably between studied cities. Such variability may be explained by specific reasons for each city. In Krasnodar located in region of warmer climate, the routine building’s operation may be less aimed at saving heat taking into account likely high outdoor temperatures throughout the year. Such situation associates with lower increase of radon concentration. Other cities are located in colder climatic zones, therefore it is assumed that the practice of operating buildings in these cities maintains the design level of energy efficiency. The highest increase of radon concentration in Ekaterinburg can be associated with a relatively lower average outdoor temperature during the measurement period. Longer uncontrolled ventilation intervals during the colder seasons may result in lower average ventilation rate in energy efficient buildings. A relatively small number of buildings with a high energy efficiency index got to the study in Chelyabinsk. The outdoor temperature during the measurement period was the highest in this city. Therefore, the conclusion about increasing of radon concentration in building of high energy efficient class requires further justification in this city.

Some deviations from the correlation between the average radon concentration and EEI may be caused by some additional factors. In particular, radon concentration is associated with the radium-226 activity concentration, emanation factor and other characteristics in building materials, which may vary for different types of buildings in the same city. This may be caused by using different types of building structures and building materials at different periods of time. E.g. in previous studies, relatively high radium-226 concentration in building materials (90 Bq/kg) was found in a flat of new energy efficient building (Ekaterinburg, 15-th floor), which, in combination with low uncontrolled ventilation rate, caused high indoor radon concentration (370 Bq/m^3^)^20^.

During the formation of samples, certain skewness towards the new buildings was permitted in order to ensure representativeness of the energy efficient buildings in the study. Therefore, the created samples may contain energy efficient buildings in higher proportion than their real contribution to the housing stock in the cities. According to the assessment of EEI based on architectural and construction characteristics, not all buildings built after 2000 have been assigned to high energy efficiency class. Thought some bias in forming samples of new buildings was allowed, a percentage of high energy efficiency buildings in the sample of new buildings in different cities may be compared. The largest percentages of energy efficient buildings among new buildings were observed in Saint Petersburg. In Chelyabinsk, considerable part of houses built in the 2000s was panel buildings with lower energy efficiency. Difference in the percentage of high energy efficiency buildings reflects preferable appliance of different construction technologies and, to some extent, the social and economic development of the cities. Higher percentage of new energy efficient buildings may result in relative increase of radon concentration in a larger number of houses in Saint Petersburg.

A detailed analysis of the radon accumulation in the new energy efficient buildings in Ekaterinburg and Saint Petersburg has shown that this group of buildings is heterogeneous. A higher radon concentration was found in the buildings built recently that may be caused by the following reasons. On the one hand, it is possible that the further improvement of technologies that raise the building's energy efficiency occurs. On the other hand, it can be assumed that after few years of putting the building into operation, its energy efficiency may reduce due to the deterioration of some structural elements of the building. Also, low ventilation and high radon accumulation may occur in flats not occupied by residents within few months after construction completing, which increase total radon activity in new flat houses. Among flats in new built buildings (after 2015), low attendance was reported in the questionnaires for 3 flats (all in Saint Petersburg). Some amount of inhabited flats in the new building may influence on total ventilation rate in a building. However the effect of advective flows between habited and inhabited premises of a multi-flat building is insufficient studied yet.

The fundamental difference should be noted between the multi-storey buildings analyzed in this study and the low-rise detached houses, which are in the spot in the studies of energy saving in Western Europe and Northern America. In low-rise buildings, the main source of radon is ground under the building^43,44^. Improving the airtightness of a building can increase the pressure difference in the soil-building system and trigger the stack effect^18,43^. This situation leads to an increase in the indoor radon concentration due to radon entry from the soil and low dilution by fresh air. In high-rise buildings, the air flow from the soil does not play a significant role in the radon entry on the upper floors, and the construction materials become a main source of radon^35–38^. Improving the airtightness of the high-rise multi-storey buildings can also cause depression in individual rooms and appearing additional convective air flows distributed between the building spaces. This potential effect needs to be studied in the future.

The number of theoretical studies and analysis of construction practices have shown that providing additional mechanical ventilation can solve the problem of air quality during thermal reconstruction of houses and the construction of new energy-efficient buildings. In this study, one house equipped with the mechanical ventilation system with heat recovery was identified in the surveyed sample of modern energy efficient multi-storey apartment buildings. In this new energy efficient building in Saint Petersburg average radon concentration was 30 Bq/m^3^, that is comparable with average radon concentration in buildings with EEI > 1 in this city. In other buildings, special engineering solutions are not used to improve energy efficiency without compromising the comfort of the indoor climate. The effectiveness of building ventilation strategies using mechanical ventilation systems in high-rise buildings in terms of radon accumulation also requires further study.

In general, observed indoor radon concentrations in multi-storey apartment houses in studied Russian cities are relatively low. Radon concentration higher than action level established in Russia for newly built houses (equilibrium equivalent radon concentration 100 Bq/m^3^ which is equal to radon concentration 200 Bq/m^3^ if equilibrium factor 0.5 is applied) was found in few apartments in energy-efficient buildings in Ekaterinburg. In other cities radon concentration was lower than this action level in all studied multi-storey houses. However, limiting the analysis to only comparing the current situation with the action level potentially hides a problem of relative increase of radon concentration in new buildings with high energy efficiency class, which requires attention from point of view of the radiological protection. The mass construction of energy-efficient buildings, which is now taking place in large cities of Russia, in the long term, can lead to an increase in mean and collective radiation doses of the urban population. Framing and implementation of the radon protection strategy should be based on the principles of justification and optimization^5^ taking into account social and economic factors and evaluation of the situation of exposure to indoor radon in different types of buildings.

## Conclusions

The novelty of this study lies in the combined survey of radon concentration in dwellings and architectural and construction characteristics with a quantitative estimation of energy efficiency potential of a building. An energy efficiency parameter (EEI) describing the energy efficiency of a building in terms of reducing uncontrolled air exchange has been proposed for the first time. A comparative analysis of results of measurements carried out in the same period of time in different types of buildings provides evidence of the relationship between the energy efficiency of a building and the indoor radon concentration in large samples of buildings in real operating conditions.

The main conclusions of the study are as follow:

1. Assumption that radon concentration in modern multi-storey apartment energy-efficient buildings is higher on average than in typical flat houses of the previous periods of construction is confirmed in four Russian cities.
2. Increased energy efficiency causing lower uncontrolled ventilation rate is a factor that reduces the radon safety of a multi-storey apartment building.
3. Energy efficiency does not predetermine the excess of reference levels and national radiation-hygienic standards in new buildings.
4. Relatively higher levels of radon concentration in energy-efficient buildings may be associated with low uncontrolled ventilation rate and multifamily building’s operation practices which caused insufficient average air exchange rate.
5. Higher activity concentration of radium-226 in building materials may enhance effect of airtightness.
6. The potential increase of radiation doses due to introduction of technologies for the construction of energy-efficient buildings should be analyzed taking into account the principle of radiological protection.
